# Spatial proteomics of hippocampal subfield‐specific pathology in Alzheimer's disease and primary age‐related tauopathy

**DOI:** 10.1002/alz.13484

**Published:** 2023-09-30

**Authors:** Jamie M. Walker, Miranda E. Orr, Timothy C. Orr, Emma L. Thorn, Thomas D. Christie, Raquel T. Yokoda, Meenakshi Vij, Alexander J. Ehrenberg, Gabriel A. Marx, Andrew T. McKenzie, Justin Kauffman, Enna Selmanovic, Thomas Wisniewski, Eleanor Drummond, Charles L. White, John F. Crary, Kurt Farrell, Tiffany F. Kautz, Elena V. Daoud, Timothy E. Richardson

**Affiliations:** ^1^ Department of Pathology Molecular and Cell‐Based Medicine Icahn School of Medicine at Mount Sinai New York New York USA; ^2^ Nash Family Department of Neuroscience Icahn School of Medicine at Mount Sinai New York New York USA; ^3^ Neuropathology Brain Bank & Research CoRE Icahn School of Medicine at Mount Sinai New York New York USA; ^4^ Glenn Biggs Institute for Alzheimer's & Neurodegenerative Diseases University of Texas Health Science Center San Antonio Texas USA; ^5^ Section of Gerontology and Geriatric Medicine Department of Internal Medicine Wake Forest School of Medicine Winston‐Salem North Carolina USA; ^6^ Sticht Center for Healthy Aging and Alzheimer's Prevention Wake Forest School of Medicine Winston‐Salem North Carolina USA; ^7^ Salisbury VA Medical Center Salisbury North Carolina USA; ^8^ Department of Healthcare Innovations Wake Forest School of Medicine Winston‐Salem North Carolina USA; ^9^ Memory and Aging Center Weill Institute for Neurosciences University of California San Francisco San Francisco California USA; ^10^ Helen Wills Neuroscience Institute University of California Berkeley Berkeley California USA; ^11^ Innovative Genomics Institute University of California, Berkeley Berkeley California USA; ^12^ Department of Artificial Intelligence & Human Health Icahn School of Medicine at Mount Sinai New York New York USA; ^13^ Ronal M. Loeb Center for Alzheimer's Disease Icahn School of Medicine at Mount Sinai New York New York USA; ^14^ Department of Neurology Icahn School of Medicine at Mount Sinai New York New York USA; ^15^ Friedman Brain Institute Icahn School of Medicine at Mount Sinai New York New York USA; ^16^ Department of Psychiatry Icahn School of Medicine at Mount Sinai New York New York USA; ^17^ Department of Pathology New York University Grossman School of Medicine New York New York USA; ^18^ Department of Psychiatry New York University Grossman School of Medicine New York New York USA; ^19^ Center for Cognitive Neurology Department of Neurology New York University Grossman School of Medicine New York New York USA; ^20^ Brain & Mind Center and School of Medical Sciences Faculty of Medicine and Health University of Sydney Sydney Australia; ^21^ Department of Pathology University of Texas Southwestern Medical Center Dallas Texas USA

**Keywords:** aging, Alzheimer's disease (AD), neurodegeneration, primary age‐related tauopathy (PART), resilience, resistance, synapses

## Abstract

**INTRODUCTION:**

Alzheimer's disease (AD) and primary age‐related tauopathy (PART) both harbor 3R/4R hyperphosphorylated‐tau (p‐tau)‐positive neurofibrillary tangles (NFTs) but differ in the spatial p‐tau development in the hippocampus.

**METHODS:**

Using Nanostring GeoMx Digital Spatial Profiling, we compared protein expression within hippocampal subregions in NFT‐bearing and non‐NFT‐bearing neurons in AD (*n* = 7) and PART (*n* = 7) subjects.

**RESULTS:**

Proteomic measures of synaptic health were inversely correlated with the subregional p‐tau burden in AD and PART, and there were numerous differences in proteins involved in proteostasis, amyloid beta (Aβ) processing, inflammation, microglia, oxidative stress, and neuronal/synaptic health between AD and PART and between definite PART and possible PART.

**DISCUSSION:**

These results suggest subfield‐specific proteome differences that may explain some of the differences in Aβ and p‐tau distribution and apparent pathogenicity. In addition, hippocampal neurons in possible PART may have more in common with AD than with definite PART, highlighting the importance of Aβ in the pathologic process.

**Highlights:**

- Synaptic health is inversely correlated with local p‐tau burden.
- The proteome of NFT‐ and non‐NFT‐bearing neurons is influenced by the presence of Aβ in the hippocampus.
- Neurons in possible PART cases share more proteomic similarities with neurons in ADNC than they do with neurons in definite PART cases.

## 1 BACKGROUND

Alzheimer's disease (AD) is the most common and extensively studied neurodegenerative disease. From a neuropathologic standpoint, the distribution of two proteinopathic lesions characterizes AD neuropathologic change (ADNC). Amyloid beta (Aβ) plaques progressively appear in the neocortex, then the hippocampus and limbic structures, followed by the brainstem and cerebellum.^1^ Hyperphosphorylated‐tau (p‐tau)‐positive neurofibrillary tangles (NFTs), on the other hand, first appear in the locus coeruleus, then subsequently involve the entorhinal/transentorhinal regions before progressing through the hippocampus proper and other limbic structures to the neocortex in well‐defined Braak stages that correspond roughly with the degree of clinical symptoms.^2^, ^3^ In the hippocampus of AD subjects, this pathology may affect any subregion, although studies have shown that the entorhinal cortex and CA1 are thought to be the most severely affected, with CA2 pathology typically first developing in Braak stage IV, and this distribution may be determined by the presence and extent of hippocampal Aβ deposition.^2^, ^4^, ^5^, ^6^, ^7^

Primary age‐related tauopathy (PART), previously termed “tangle‐only senile dementia” or “age‐related neurofibrillary degeneration,” is an Aβ‐independent accumulation of neurofibrillary degeneration in medial temporal lobe structures.^8^, ^9^ Similarly to ADNC, the NFTs found in PART are composed of paired helical p‐tau filaments comprising both 3R and 4R tau isoforms,^5^, ^9^ which are indistinguishable by cryo‐EM analysis from the NFTs found in ADNC subjects.^10^ The p‐tau pathology of PART is confined mainly to the locus coeruleus, entorhinal cortex, and hippocampal subregions, with minor extension into the neocortex in rare cases,^5^, ^9^, ^11^ and recent longitudinal neuroimaging data suggest that PART‐associated p‐tau pathology generally does not progress into the neocortex or “convert” into AD.^12^ Neurofibrillary degeneration in PART also displays an early selective predilection for the CA2 hippocampal subregion, unlike in ADNC, where this region is typically spared until later in the disease process.^4^, ^5^, ^6^, ^7^ Definite PART is defined as Braak stage I‐IV in the complete absence of Aβ deposition (Thal phase 0 and CERAD neuritic plaque [NP] score “absent”), while possible PART is defined as Braak stage I‐IV with minimal Aβ deposition, below that which would typically qualify for a designation of ADNC (Thal phase 1 or 2 and CERAD NP score “absent” or “sparse”).^5^, ^9^

TAR DNA‐binding protein 43 (TDP‐43) Unlike in AD, Braak stage does not appear to correlate well with cognitive function in subjects with PART.^5^, ^13^ Cognitive status in PART subjects has been shown to be related to the overall hippocampal p‐tau burden,^6^, ^13^, ^14^, ^15^, ^16^ as well as the presence of additional comorbid neuropathologic findings, including limbic‐predominant age‐related TAR DNA‐binding protein of 43 kDA (TDP‐43) encephalopathy (LATE), Lewy body disease (LBD), aging‐related tau astrogliopathy (ARTAG), cerebrovascular disease (CVD), and white matter abnormalities.^15^, ^17^, ^18^, ^19^ Patients with “pure” PART (ie, those without other known neuropathologic comorbidities) do not appear to have impaired global cognition compared to age‐matched individuals with no neurodegenerative changes identified at autopsy; however, more specific neuropsychological testing has identified deficits in visuospatial function, executive function, and processing speed, suggesting that these cognitive domains may be influenced by CA2 subregion pathology.^15^, ^19^ In AD, synaptic loss is associated with increasing p‐tau burden and thought to underlie cognitive impairment,^20^, ^21^, ^22^ and recent work identified synaptic loss in the CA2 subregion with increasing hippocampal p‐tau burden in individuals with PART.^23^

Currently, little is known about proteomic differences between NFT‐bearing neurons in AD and PART, how those may affect the hippocampal p‐tau distribution, or the role that Aβ burden and distribution may play in this process. In this study, we utilize Nanostring GeoMx Digital Spatial Profiling (DSP) to compare proteomic differences in both NFT‐bearing neurons and non‐NFT‐bearing neurons (“normal neurons”) across the entorhinal cortex, CA1, and CA2 hippocampal subregions between subjects with AD, definite PART, and possible PART. We investigate the subregional health of synapses and expression levels of proteins involved in proteostasis, Aβ and tau processing, oxidative stress, inflammation, and gliosis between disease states and in relation to subregional p‐tau burden and Aβ progression.

## 2 METHODS

### 2.1 Case selection

Fourteen subjects with brain tissue stored in the Mount Sinai Neuropathology Brain Bank and Research CoRE, collected between 2016 and 2022, were evaluated. The neuropathologic diagnoses given at the time of original autopsy evaluation, hematoxylin and eosin (H&E), and immunohistochemical (IHC) stains were re‐reviewed by two board‐certified neuropathologists (J.M.W. and T.E.R.) to ensure consistency in diagnoses. These cases included seven subjects with ADNC (two with intermediate‐level ADNC and five with high‐level ADNC) and seven subjects with PART (three with definite PART and four with possible PART) (Table 1). By design, all cases evaluated (ADNC, definite PART, and possible PART) had some degree of p‐tau‐positive neurofibrillary degeneration, defined here as at least five NFTs available for proteomic analysis, in each of the evaluated regions (entorhinal cortex, CA1, and CA2).

**RESEARCH IN CONTEXT**

1. **Systematic review**: The authors reviewed the literature using online journal sources. The proteomics of AD and primary age‐related tauopathy (PART) have been studied, but the spatial proteomics in AD and PART within specific hippocampal subregions have not.
2. **Interpretation**: Although it is known that Aβ and p‐tau interact at the level of the hippocampus, it is unclear how this interaction affects other proteins in NFT‐ and non‐NFT‐bearing neurons. Proteomic differences that are specific to disease state, neuron type, and subregion and related to Aβ progression are demonstrated. These results indicate that possible PART may have more in common with AD than with definite PART.
3. **Future directions**: The described proteomic findings could be used as a basis for further multi‐omic studies and to develop diagnostic biomarkers. This study provides mechanistic insight examining ways by which individuals with PART are able to evade plaque deposition.

**TABLE 1:** Clinical and pathologic features of ADNC and PART cases.

			ADNC Level					
Case	Age	Gender	Braak stage	Thal phase	CERAD NP Score	Primary diagnosis	Additional diagnoses	Cognitive status
1	83	F	VI (B3)	5 (A3)	Frequent (C3)	ADNC, high‐level	CAA, CVD	Clinical dementia
2	74	M	VI (B3)	4 (A3)	Frequent (C3)	ADNC, high‐level	CVD	Clinical dementia
3	83	F	V (B3)	4 (A3)	Moderate (C2)	ADNC, high‐level	Remote infarct, right cerebellum	Clinical dementia
4	77	F	V (B3)	4 (A3)	Moderate (C2)	ADNC, high‐level	Subarachnoid hemorrhage	Clinical dementia
5	91	F	V (B3)	4 (A3)	Frequent (C3)	ADNC, high‐level	–	Clinical dementia
6	60	M	IV (B2)	3 (A2)	Moderate (C2)	ADNC, intermediate‐level	CVD	Unknown
7	82	M	IV (B2)	3 (A2)	Moderate (C2)	ADNC, intermediate‐level	CAA	Unknown
8	81	M	IV (B2)	0 (A0)	None (C0)	PART, definite	CAA, CVD	Clinical dementia
9	75	M	III (B2)	0 (A0)	None (C0)	PART, definite	ARTAG, LATE‐NC	Clinical dementia
10	85	M	III (B2)	0 (A0)	None (C0)	PART, definite	–	Non‐demented
11	85	M	IV (B2)	2 (A1)	None (C0)	PART, possible	–	Mild cognitive Impairment
12	73	F	IV (B2)	2 (A1)	None (C0)	PART, possible	CAA, ARTAG	Non‐demented
13	78	F	IV (B2)	2 (A1)	None (C0)	PART, possible	CAA, CVD	Non‐demented
14	71	F	III (B2)	2 (A1)	None (C0)	PART, possible	–	Non‐demented

Abbreviations: ADNC, Alzheimer's disease neuropathologic change; ARTAG, aging‐related tau astrogliopathy; CAA, cerebral amyloid angiopathy; CVD, cerebrovascular disease; LATE‐NC, limbic‐predominant age‐related TDP‐43 encephalopathy neuropathologic change; PART, primary age‐related tauopathy.

### 2.2 Immunohistochemistry

Standard histologic and IHC workups were performed for complete diagnostic phenotyping in each case. For this study, 5‐μm‐thick formalin‐fixed paraffin‐embedded (FFPE) sections of the posterior hippocampus (level 7)^24^ were mounted on charged slides and baked at 70°C. H&E, Bielschowsky silver stain, p‐tau IHC (AT8; MN1020, Thermo Fisher Scientific), and Aβ IHC (6E10; SIG‐39320, Covance, Inc.) were performed on all sections using a Leica Bond III automated immunostainer (Leica Biosystems), according to the manufacturer's protocols.

### 2.3 Computer‐assisted assessment of p‐tau

Hippocampal subfields (CA1 and CA2) and entorhinal cortex were segmented using Aperio ImageScope software, as previously described.^6^, ^13^, ^25^ The CA2 hippocampal subfield was defined neuroanatomically as the most compact region of cornu ammonis neurons. The CA1 subfield was defined as the region between the distal CA2 boundary and the hippocampal fissure. Each region was outlined with the Aperio ImageScope pen tool. P‐tau burden was determined using Aperio ImageScope positive pixel count version nine default parameters (intensity threshold for weak positive pixels [upper limit] = 220, intensity threshold for weak positive pixels [lower limit] = 175, intensity threshold for medium positive pixels [lower limit] = 100, intensity threshold for strong positive pixels [lower limit] = 0) (Figure S1). The sum of medium and strong positive pixels was divided by total pixels to establish a 0‐1 ratio of positive pixels/total pixels (positive pixel proportion; PPP), with validation performed as previously described.^6^, ^13^, ^25^

### 2.4 NanoString GeoMxTM DSP

**FIGURE 1:**
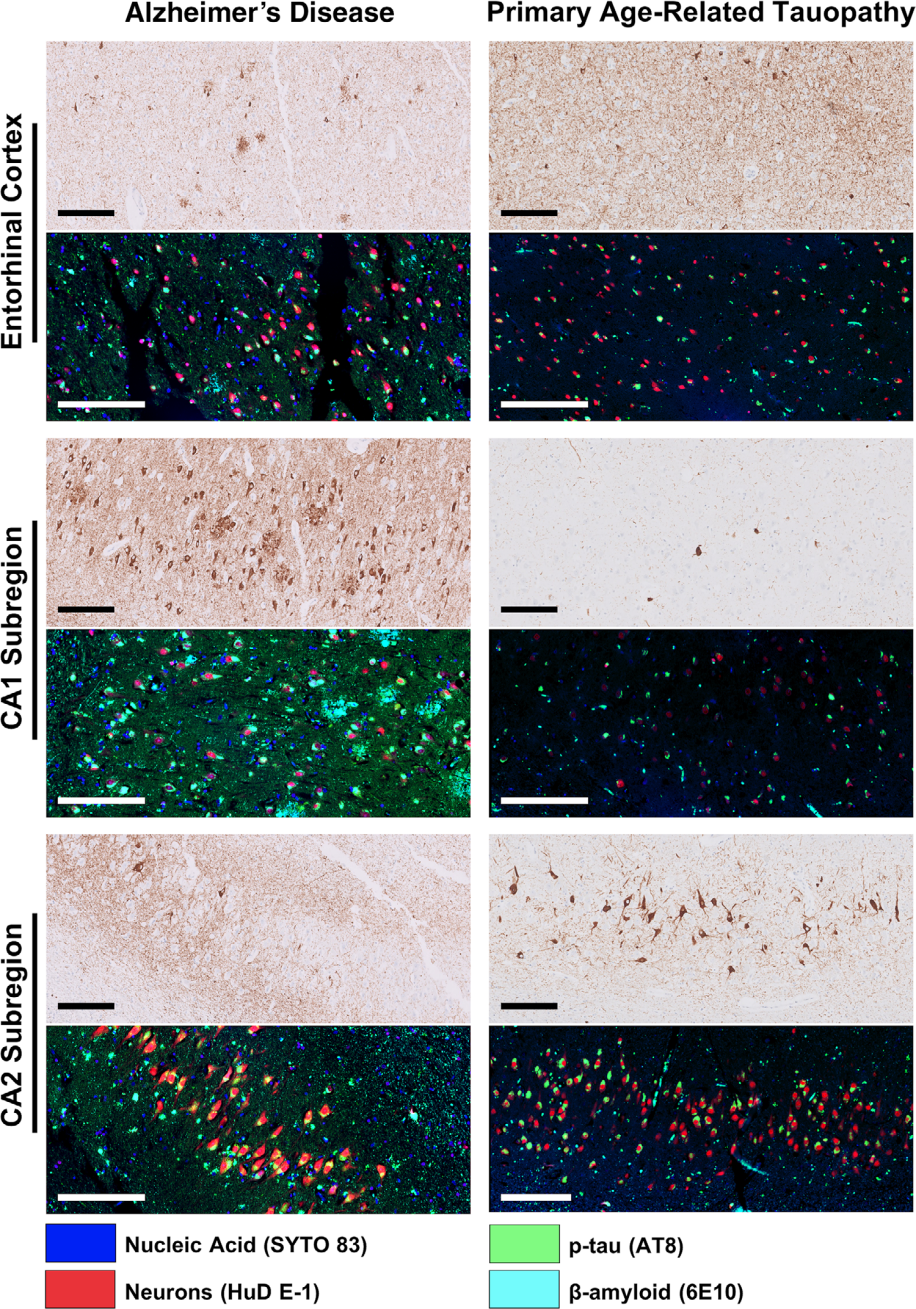
Representative paired p‐tau (AT8) immunohistochemical stains and immunofluorescent stains demonstrating p‐tau and Aβ pathology in entorhinal cortex, CA1 subregion, and CA2 subregion in a case of Alzheimer's disease (case 1) and primary age‐related tauopathy (case 8). Scale bars in all panels = 200 μm.

FFPE hippocampal sections of the posterior hippocampus (level 7)^24^ were deparaffinized and incubated with morphology markers, specific fluorescently labeled antibodies to aid in selecting regions of interest (ROIs), according to the manufacturer's instructions using protocols specifically developed for DSP assays (https://nanostring.com/products/geomx‐digital‐spatial‐profiler/geomx‐protein‐assays/). Here, NFTs were labeled with a fluorescently labeled antibody against AT8 (phospho‐tau, Ser202, and Thr205, conjugated with an Alexa Fluor 594 [AF594] antibody labeling kit from Thermo Fisher Scientific), Aβ (6E10; conjugated with an Alexa Fluor 488 [AF488] antibody labeling kit from Thermo Fisher Scientific), and neuronal nuclei  (HuD E‐1; Alexa Fluor 647; Thermo Fisher Scientific). Nucleic acid was identified by staining with SYTO 83 (Thermo Fisher Scientific) (Figure 1). Each ROI consisted of a 50‐μm‐diameter circle (1963.5 μm^2^ surface area) centered on the cell body/nucleus of a single neuron, encompassing the neuron itself as well as its immediate microenvironment (Figure S2), a modification of our previous methodology.^26^ In each case, five individual NFT‐bearing neurons and five non‐NFT‐bearing, AT8‐negative neurons (“normal neurons”) were selected by J.M.W. and T.E.R. for high‐resolution multiplex proteomic profiling from each hippocampal subregion (CA1 and CA2) and entorhinal cortex, for a total of 30 analyzed ROIs per slide. This resulted in a total of 35 NFT‐bearing neurons and 35 non‐NFT‐bearing neurons in each subregion of PART (210 ROIs total) and 35 NFT‐bearing neurons and 35 non‐NFT‐bearing neurons in each subregion of ADNC (210 ROIs total).

Each slide was also incubated with a cocktail containing 76 antibodies conjugated to a unique UV‐photocleavable oligonucleotide tag (Table S1). All ROIs were analyzed using NanoString's GeoMx™ Digital Spatial Profiler System (NanoString Technologies, Seattle, WA, USA).^26^, ^27^ Selected ROIs were individually illuminated with UV light, cleaving the oligonucleotides from the antibodies bound to their respective proteins in each ROI. Oligonucleotides were collected in a 96‐well plate and hybridized to four‐color, six‐spot optical barcodes and analyzed on NanoString's nCounter platform, resulting in the quantification of each antibody present in each ROI. These digital counts were normalized by a signal‐to‐noise ratio (SNR) using mouse IgG2a and rabbit IgG. Internal control housekeeper proteins (Histone H3, S6, GAPDH) have been shown to display altered expression in NFTs in AD and thus were not used for normalization.^28^, ^29^ ROIs were manually re‐inspected for accuracy after nCounter analysis using both the morphology and quantitative markers; ROIs containing NFTs were checked to ensure higher levels of p‐tau compared to ROIs containing non‐NFT‐bearing neurons.

### 2.5 Statistics

All statistical analysis was performed using GraphPad Prism version 9 (GraphPad Software, Inc., La Jolla, CA, USA). Correlations between protein quantification and p‐tau burden and Thal phase were made using linear regression modeling using Pearson's correlation coefficient. Differences between protein levels were evaluated using multiple *t* tests. Statistical significance was set at *α* = 0.05. All comparisons were corrected, and false discovery rate (FDR) correction was used for multiple comparison testing. All statistically significant differences shown throughout were significant after FDR correction.

## 3 RESULTS

### 3.1 Patient demographics and distribution of hippocampal p‐tau and Aβ

The mean age at death of the ADNC cohort was 78.6 years (range: 60 to 91 years) and the mean age of the PART cohort was 78.3 years (range: 71 to 85 years; *p* = 0.9477). Definite PART cases had a mean age of 80.3 years (range: 81 to 85 years; *p* = 0.4413), while possible PART cases had a mean age of 76.8 years (range: 71 to 85 years; *p* = 0.9010). No significant differences were noted in gender or ethnicity. The Braak stage,^2^ Thal phase,^1^ and CERAD NP Score,^30^ additional neuropathologic diagnoses including cerebral amyloid angiopathy (CAA), CVD, infarcts, hemorrhage, ARTAG, and LATE, and cognitive status are described in Table 1. No significant differences were identified between ADNC and PART cohorts in terms of these additional neuropathologic findings, including incidence or severity of LATE,^17^, ^19^, ^31^, ^32^ LBD,^19^, ^32^ or CVD.^19^, ^32^, ^33^

None of the PART cases displayed NP, while all ADNC cases had at least moderate frequency of NP by CERAD criteria.^30^ Three PART cases had no evidence of Aβ deposition in the neocortex or hippocampus (definite PART), while four PART cases (possible PART) had focal Aβ present in the entorhinal cortex or CA1 subregion (Thal phase 2). In contrast, all evaluated ADNC cases had at least Thal phase 3, so they had concomitant Aβ and NFTs in the entorhinal cortex and CA1 subregion, but not the CA2 subregion. All PART and ADNC cases were at least Braak stage III and had some degree of NFT deposition in each of the evaluated hippocampal subregions. Similar to our previous studies,^5^, ^6^ there was a non‐significant trend toward a higher overall p‐tau burden across the analyzed hippocampal subregions in ADNC cases (0.22 ± 0.03 PPP) compared to all included PART cases (0.11 ± 0.02 PPP; *p* = 0.0674) and definite PART cases (0.08 ± 0.02 PPP; *p* = 0.0766). There was a significantly lower CA2/CA1 ratio in ADNC cases (0.57 ± 0.16) compared to all PART cases (1.78 ± 0.29; *p* = 0.0109) and definite PART cases (2.18 ± 0.47; *p* = 0.0151). This latter measure is important, as previous studies indicated that ADNC subjects typically began to develop CA2 p‐tau‐positive neurofibrillary degeneration in Braak stage IV‐VI, after more significant involvement of the entorhinal cortex and CA1 subregion, while PART subjects developed CA2 NFTs earlier and often with greater severity than other subregions,^4^, ^5^, ^6^, ^7^, ^34^ and the current cohort is consistent with those previous findings.

### 3.2 General proteomic differences between NFT‐bearing neurons and non‐NFT‐bearing neurons

Across hippocampal regions and pathologies, NFT‐bearing neurons and their immediate microenvironments contained significantly higher levels of total tau (*p* = 0.0161), p‐tau (S199) (*p* = 0.0108), p‐tau (S214) (*p* < 0.0001), p‐tau (S396) (*p* < 0.0001), and p‐tau (S404) (*p* < 0.0001), but not p‐tau (T231) (*p* = .1135), as compared to non‐NFT‐bearing neurons. Compared to non‐NFT‐bearing neurons and their immediate microenvironments, NFT‐bearing neurons and their immediate microenvironments also displayed higher levels of proteins involved in autophagy and protein degradation, including p62 (*p* < 0.0001), cathepsin D (CTSD; *p* = 0.0055), and GPNMB (*p* = 0.0011), as well as proteins involved in Aβ processing, including neprilysin (*p* < 0.0001).

### 3.3 Synaptic and dendritic integrity are inversely correlated with local/subregional p‐tau burden

**FIGURE 2:**
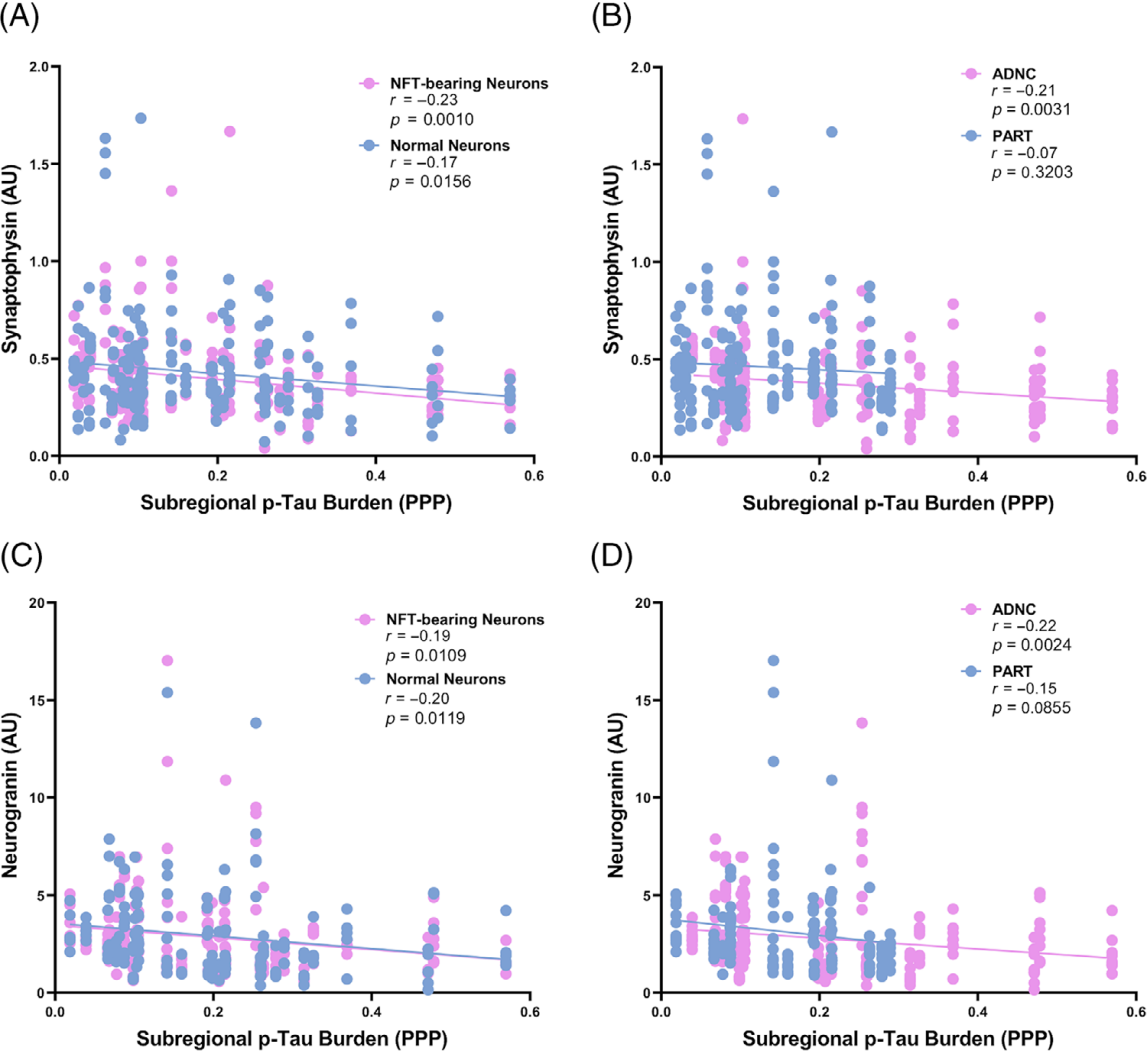
Correlation of presynaptic and postsynaptic proteins with local (subregional) p‐tau burden in all hippocampal subregions. There is a significant inverse relationship between the local p‐tau burden and synaptophysin in both NFT‐bearing neurons and non‐NFT‐bearing neurons (“normal neurons”) and the immediate microenvironments surrounding these neurons (A). This relationship is significant in ADNC cases but not in PART cases (B). Similarly, there is a significant inverse relationship between the local p‐tau burden and neurogranin in both NFT‐bearing neurons and normal neurons and the immediate microenvironments surrounding these neurons (C). This relationship is significant in ADNC cases but only reaches the trend level in PART cases (D).

To assess the role of localized tau burden, we correlated the DSP levels of the well‐established presynaptic protein, synaptophysin,^21^ and a dendritic/postsynaptic protein, neurogranin^35^ associated with both NFT‐bearing neurons and non‐NFT‐bearing neurons in each subregion to the amount of p‐tau present in each subregion (determined by Aperio ImageScope positive pixel quantification in the entorhinal cortex and the CA1 and CA2 hippocampal subregions, as previously described^6^, ^13^, ^25^) (Figure S1). There was a significant inverse correlation between synaptophysin and p‐tau burden in both NFT‐bearing neurons (*r* = −0.23; *p* = 0.0010) and non‐NFT‐bearing neurons (*r* = −0.17; *p* = 0.0156) in all subregions (Figure 2A). This was significant in ADNC NFTs and normal neurons (*r* = −0.21; *p* = 0.0031) but not in PART (*r* = −0.07; *p* = 0.3203) (Figure 2B). Similarly, there was a significant inverse correlation between neurogranin and p‐tau in both NFT‐bearing neurons (*r* = −0.19; *p* = 0.0109) and non‐NFT‐bearing neurons (*r* = −0.20; *p* = 0.0119) (Figure 2C). As with synaptophysin, there was a significant inverse relationship between neurogranin and subregional p‐tau burden in ADNC (*r* = −0.22; *p* = 0.0024), although this only approached the trend level in PART (*r* = −0.15; *p* = 0.0855) (Figure 2D). These data suggest that healthy synapses (as measured by the pre‐ and postsynaptic proteins synaptophysin and neurogranin) are lost in and in the immediate microenvironment of both NFT‐ and non‐NFT‐bearing neurons as the local p‐tau burden increases, although this relationship is only significant in ADNC neurons.

### 3.4 Proteomic differences between NFT‐bearing neurons in PART and ADNC

**FIGURE 3:**
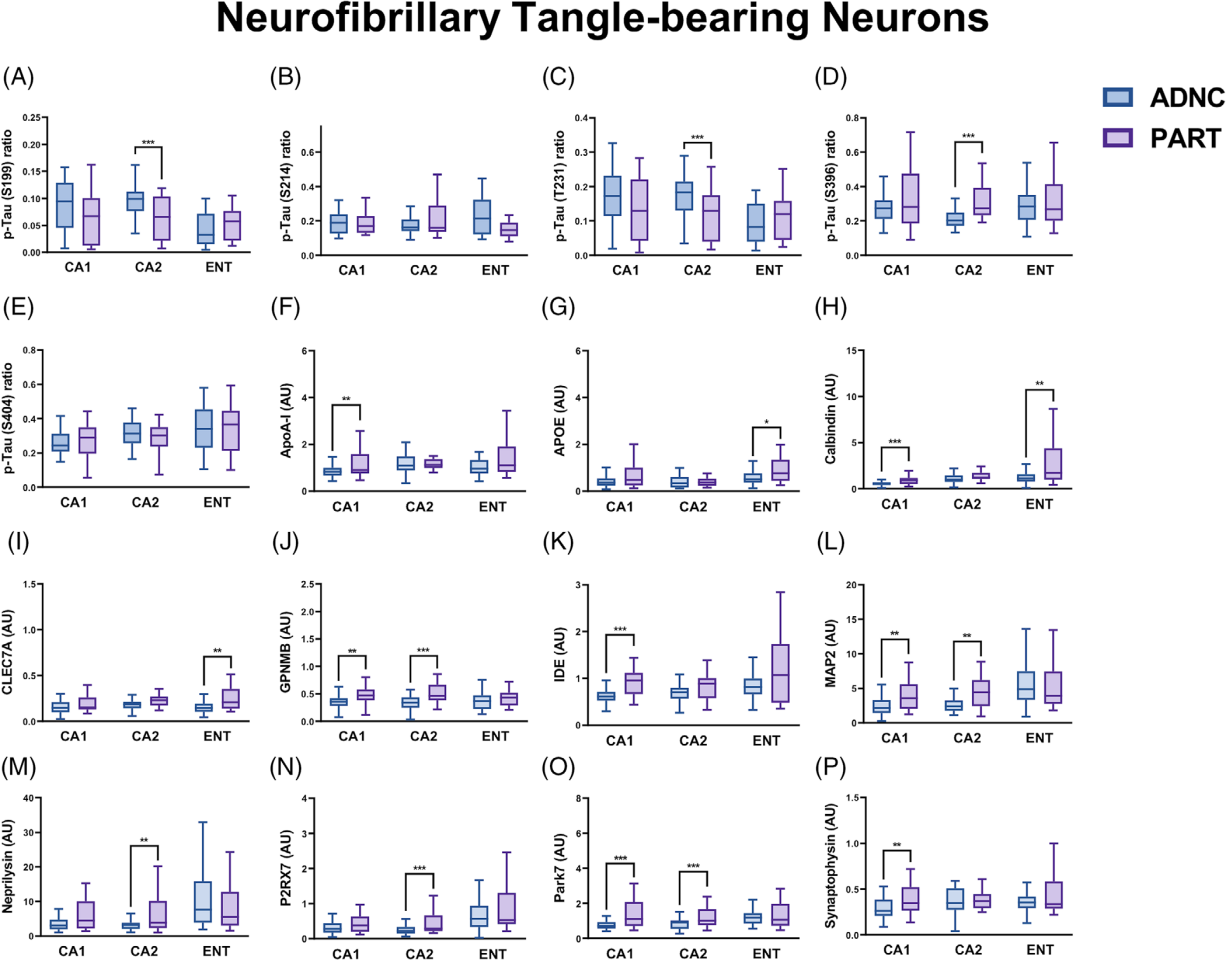
Significant differences in protein levels in NFT‐bearing neurons (and their immediate microenvironments) between ADNC and PART in each hippocampal subregion evaluated. ^*^ *p* < 0.05; ^**^ *p* < 0.01; ^***^ *p* < 0.001.

Direct comparison of NFT‐bearing neurons (and their respective immediate microenvironments) in ADNC and PART cases demonstrated lower levels of p‐tau phosphorylated at the S199 and T231 residues, as well as higher levels of p‐tau phosphorylated at S396 in the CA2 subregion of PART compared to AD without any additional differences in the CA1 or entorhinal subregions, or differences in p‐tau phosphorylated at the S214 or S404 residues (Figure 3A–E). In addition, there were significant differences in proteins associated with Aβ processing (ApoA‐I, APOE, IDE, neprilysin), inflammation, microglial function, and autophagy (CLEC7A, GPNMB, P2RX7), oxidative stress (Park7), and neuronal/synaptic health (synaptophysin, calbindin, and MAP2) between PART and AD (Figure 3F–P). In most cases, these proteomic differences appeared to be subregion dependent. For example, synaptophysin was lower in the CA1 subregion in ADNC as compared to PART, where AD displays a higher p‐tau burden. However, in the CA2 subregion (where PART tends to develop the highest tangle burden), synaptophysin levels were statistically equivalent when comparing PART and AD.^5^, ^6^, ^23^ The same pattern was true for calbindin expression in these subregions.

**FIGURE 4:**
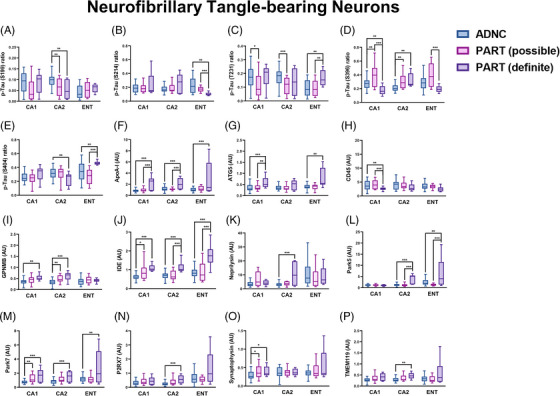
Significant differences in protein levels in NFT‐bearing neurons (and their immediate microenvironments) between ADNC, definite PART, and possible PART in each hippocampal subregion evaluated. ^*^ *p* < 0.05; ^**^ *p* < 0.01; ^***^ *p* < 0.001.

Given that the current definition of PART includes a spectrum of pathology with some cases completely devoid of Aβ and some cases having Aβ restricted to Thal phase 1 and 2 with absent or sparse NP,^5^, ^9^ we divided the PART cohort into definite and possible PART subgroups. Significant differences between definite and possible PART were found in multiple p‐tau epitopes, including S214, T231, S396, and S404, in which the p‐tau profile of possible PART was more similar to ADNC than to definite PART in many hippocampal subregions (Figure 4A–E). Additionally, there were significant differences between definite and possible PART in terms of protein degradation and autophagy‐related proteins (ATG5, GBA, GPNMB, Park5), Aβ processing (ApoA‐I, IDE, neprilysin), the complement system, inflammation, and microglia (C4B, CD45, IBA, GPNMB, MERTK, P2RX7, TMEM119), protection against oxidative stress (Park7), and neuronal/synaptic health (synaptophysin), with possible PART being more similar to ADNC than to definite PART in many proteins and subregions (Figure 4F–P and Figure S3). Importantly, these findings suggest that definite PART, which has no identifiable Aβ on routine diagnostic IHC staining, has increased levels of proteins related to Aβ degradation compared to both ADNC and possible PART (both of which have Aβ in the entorhinal and CA1 subfield). As expected, definite and possible PART also appeared to have healthier synapses in the CA1 subregion compared to ADNC, and ADNC and possible PART had higher general inflammatory markers in CA1, while definite PART had higher microglial markers, particularly in the CA2 subregion. Definite PART also harbored higher levels of Park7, which is a protein that plays an important role in cellular protection against oxidative stress.^36^, ^37^

Grouping protein expression by subregion within diseases demonstrated differential p‐tau epitope levels between hippocampal subregions in ADNC only (Figure S4A–E). ADNC displayed lower levels of synaptophysin in the CA1 subregion compared to CA2 and entorhinal cortex, as well as lower CTSD, glial fibrillary acidic protein, MER Proto‐Oncogene, Tyrosine Kinase (MERTK), neprilysin, neurogranin, P2RX7, Park5, Park7, and S100B in the CA1 and CA2 subregions compared to the entorhinal cortex. Interestingly, p62 was lowest in CA2 in ADNC and Aβ1‐42 was specifically decreased in the CA2 subregion of PART without any significant differences between subregions in ADNC (Figure S4F–S).

### 3.5 Proteomic differences between non‐NFT‐bearing neurons (“normal neurons”) in PART and ADNC

**FIGURE 5:**
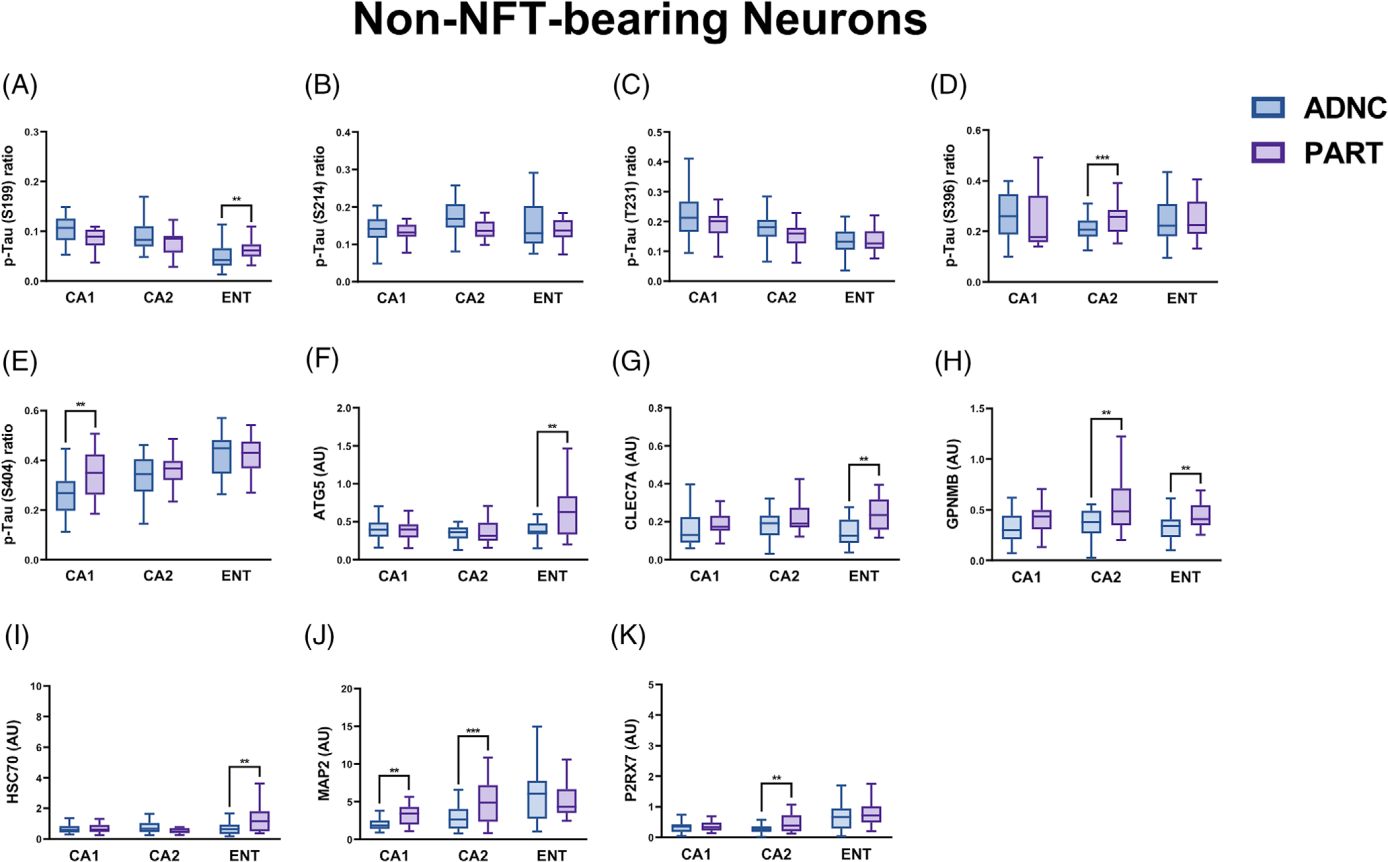
Significant differences in protein levels in non‐NFT‐bearing neurons (and their immediate microenvironments) between ADNC and PART in each hippocampal subregion evaluated. ^*^ *p* < 0.05; ^**^ *p* < 0.01; ^***^ *p* < 0.001.

**FIGURE 6:**
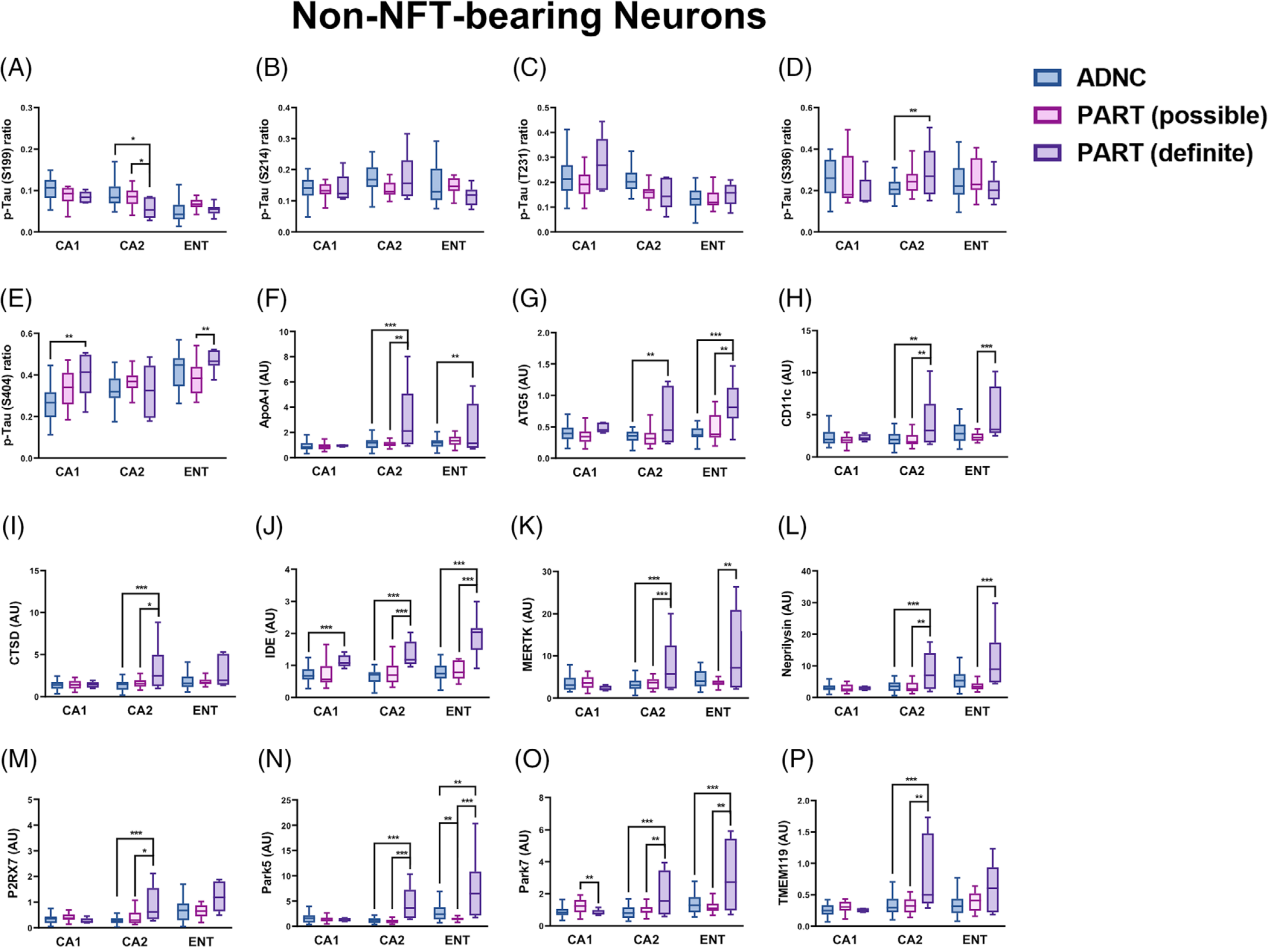
Significant differences in protein levels in non‐NFT‐bearing neurons (and their immediate microenvironments) between ADNC, definite PART, and possible PART in each hippocampal subregion evaluated. ^*^ *p* < 0.05; ^**^ *p* < 0.01; ^***^ *p* < 0.001.

Comparing non‐NFT‐bearing “normal neurons” between ADNC and all PART cases demonstrated fewer differences in terms of p‐tau epitopes (Figure 5A–E) but significant differences in ATG5 (autophagy), CLEC7A (microglia and inflammation), GPNMB (microglia and protein degradation/autophagy pathways), HSC70 (protein degradation/autophagy), MAP2 (neuronal health), and P2RX7 (microglia) (Figure 5F–K and Figure S3). After dividing the PART cohort into possible and definite PART, differences were noted in p‐tau epitopes between ADNC, possible PART, and definite PART (Figure 6A‐E). Similar differences were identified here as with NFT‐bearing neurons. Normal neurons in definite PART displayed increased levels of proteins involved in Aβ processing (ApoA‐I, IDE) compared to both possible PART and ADNC, particularly in the CA2 subregion. There were also generally higher levels of proteins involved in autophagy and protein degradation (ATG5, CTSD, HSC70, Park5), inflammation and microglia markers (CD11c, GPNMB, MERTK, P2RX7, TMEM119), and protection against oxidative stress (Park7) in definite PART compared to possible PART and ADNC, particularly in the entorhinal cortex and CA2 subregion (Figure 6F–P and Figure S3). Numerous differences in p‐tau epitopes and other proteins were identified between subregions within disease states in non‐NFT‐bearing neurons as well (Figure S5). Similar to NFT‐bearing neurons, the proteomic differences identified between ADNC, possible PART, and definite PART in non‐NFT‐bearing neurons suggest altered autophagy, inflammation, oxidative stress response, and tau/Aβ processing between ADNC and definite PART, whereas possible PART had a proteome profile more similar to ADNC in many instances.

### 3.6 Correlations between Thal phase and proteome of NFT‐bearing neurons and non‐NFT‐bearing neurons (“normal neurons”) in PART and ADNC

**FIGURE 7:**
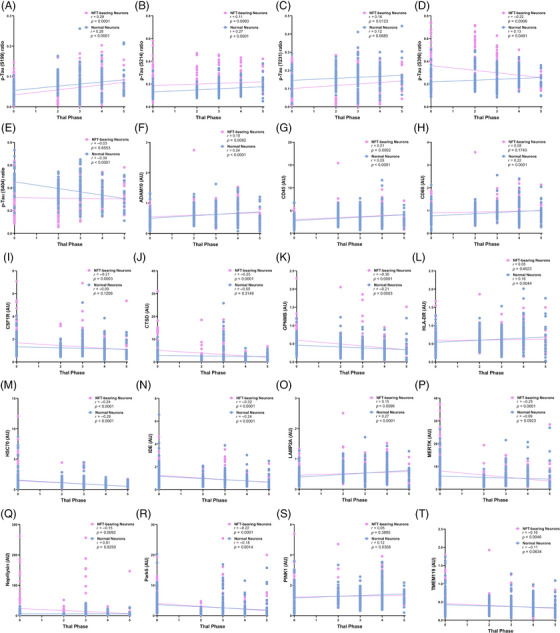
Correlation of protein levels with Thal phase in both NFT‐bearing neurons and non‐NFT‐bearing neurons (“normal neurons”) and the immediate microenvironments surrounding these neurons.

Given the observed differences between possible PART and definite PART, and to better understand the role of Aβ as a continuum, we performed linear regression analyses to investigate the correlation of Thal phase with protein expression levels in NFT‐bearing neurons and normal neurons. P‐tau epitopes generally increased with increasing Thal phase; however, interestingly, p‐tau S396 decreased in NFT‐bearing neurons, yet it increased in normal neurons, and p‐tau S404 decreased in normal neurons while remaining constant with increasing Thal phase in NFT‐bearing neurons (Figure 7A–E). In NFT‐bearing neurons there are direct relationships between Thal phase and ADAM10 (Aβ processing), CD45 (inflammation), and LAMP2A (protein degradation/autophagy), as well as inverse relationships between Thal phase and ApoA‐I (Aβ processing), CLEC7A (inflammation), CSF1R (inflammation), CTSD (protein degradation/autophagy), GPNMB (microglia and autophagy), HSC70 (protein degradation/autophagy), IBA1 (inflammation), IDE (Aβ processing), MERTK (inflammation), neprilysin (Aβ processing), Park5 (Parkinson's disease as well as a potential role in protein degradation and autophagy), and TMEM119 (microglia). In non‐NFT‐bearing neurons, there were progressive increases in ADAM10, APP (Aβ processing), CD45, CD68 (inflammation), HLA‐DR (inflammation), LAMP2A, and PINK1 (oxidative stress) with Thal phase and decreases in GPNMB, HSC70, IDE, and Park5. These changes suggest differences in inflammation and microglial function, neuronal health, Aβ processing, and oxidative stress across both NFT‐bearing and normal neurons that are affected by Aβ burden and that pathologic changes (particularly inflammatory changes) are occurring in and around hippocampal neurons before detectable NFTs develop (Figure 7F–T and Figure S3).

## 4 DISCUSSION

Although there remains disagreement over the nature of PART and its relationship to ADNC, many studies have identified clinical, molecular, and neuropathologic differences between the two entities.^38^, ^39^ In AD, p‐tau‐positive NFTs appear in the entorhinal cortex and hippocampus proper before involving the neocortex,^2^ while Aβ deposition appears first in the neocortex and progresses through the hippocampus before moving to deeper brain nuclei, the brainstem, and cerebellum.^1^ As such, the hippocampus is the neuroanatomic location where Aβ plaques and NFTs coexist for the first time in the presumed pathogenic sequence of AD, and previous DSP studies demonstrated a proteomic interaction between these two protein deposits, with possible implications for cognitive function.^26^ By definition, PART involves the medial temporal lobe with Alzheimer‐type p‐tau‐positive neurofibrillary degeneration that generally does not progress beyond the hippocampus with minimal or no involvement by Aβ,^5^, ^6^, ^9^, ^11^ making the hippocampus an ideal location to study differences between these two entities as well as the impact of Aβ on p‐tau and other proteins.^40^

By leveraging diagnoses of ADNC, definite PART, and possible PART, as well as subregional p‐tau levels and Thal phase (as a surrogate for the severity of Aβ pathology), we were able to investigate the differences between the proteome of ADNC and PART at the level of NFT‐bearing and non‐NFT‐bearing neurons, as well as their respective immediate microenvironments. At lower Aβ levels, there are higher levels of many proteins associated with protein degradation and autophagy, including CTSD,^41^, ^42^ GPNMB,^43^, ^44^ HSC70,^45^, ^46^, ^47^ and Park5,^48^, ^49^ whereas only LAMP2A^47^ displays higher expression levels with increasing Aβ (Figure 7). Similarly, NFT‐bearing neurons in definite PART have higher levels of ATG5^50^, ^51^ in CA1 and entorhinal and higher GBA in CA2 compared to ADNC and possible PART, while non‐NFT‐bearing neurons in definite PART have higher ATG5 in CA2 and entorhinal, higher CTSD in CA2, and higher HSC70 in entorhinal compared to ADNC and possible PART (Figures 4 and 6). This suggests that these proteins involved in proteostasis may be assisting in regulating the levels of aggregated proteins, including Aβ in these cases. Some proteins associated with inflammation and microglia are correlated with higher Thal phase, including CD45, CD68 (around normal neurons only), and HLA‐DR (around normal neurons only), while others, including CLEC7A^52^, ^53^ (around NFT‐bearing neurons), CSF1R^54^, ^55^ (around NFT‐bearing neurons), IBA1^56^ (around NFT‐bearing neurons), MERTK^55^, ^57^ (around NFT‐bearing neurons), and TMEM119^58^ (around NFT‐bearing neurons), are lower in cases with a higher Aβ burden. The more general markers of inflammation that increase with increasing Aβ levels (particularly around normal neurons) may indicate a localized pro‐inflammatory, deleterious state, while some of the microglial markers that are thought to be protective,^55^ including MERTK and CSF1R, are decreased (particularly around NFT‐bearing neurons) at higher Aβ levels, which may indicate a loss of a healthy response to Aβ and other misfolded proteins, similar to the decrease in proteostasis‐related proteins. In terms of proteins related to oxidative stress, PINK1 is increased in cases with higher Aβ levels, whereas Park7 is elevated in definite PART compared to ADNC and possible PART in all neurons and subregions. PINK1 is upregulated in the setting of oxidative stress, while Park7 may protect against oxidative stress, through mechanisms such as increasing SOD1.^36^, ^37^ This suggests that individuals with PART may be more capable of dealing with oxidative stress.

Proteins related to Aβ processing also demonstrate a varied response to the presence of increasing Thal phase. With higher Aβ levels, there is an increase in ADAM10, which is involved in non‐amyloidogenic amyloid precursor protein (APP) processing,^59^ and an increase in Aβ1‐40 and APP in the non‐NFT‐bearing neurons. However, the majority of Aβ‐processing proteins, including ApoA‐I, IDE, and neprilysin,^60^, ^61^, ^62^ are higher in cases with a lower Thal phase (Figure 7 and Figure S3) and are higher in definite PART compared to possible PART and ADNC (Figures 4 and 6). This suggests that definite PART cases may maintain healthy Aβ processing systems, either effectively degrading Aβ or preventing its accumulation. Finally, markers of synaptic health, synaptophysin and neurogranin, are inversely correlated with the burden of p‐tau in their respective subregions, a finding previously demonstrated in both PART and AD.^21^, ^23^ We identified decreases in both of these synaptic proteins in NFT‐bearing and non‐NFT‐bearing neurons with increasing p‐tau burden; however, this was only significant in ADNC cases (Figure 2). This may indicate that the p‐tau burden is more detrimental to synaptic health in ADNC, or it may underscore the impact of Aβ interaction with NFTs at the level of the hippocampus.

We previously investigated differences in CA1 neurons in cognitively impaired and cognitively normal (“resilient”) individuals with ADNC using DSP.^26^ Compared to demented ADNC subjects, resilient subjects maintained significantly higher levels of proteins associated with neuronal integrity, including synaptophysin and neurofilament light chain, in the NFTs and microenvironments of NFT‐bearing neurons, as well as higher levels of Park5, a ubiquitin hydrolase with a role in the degradation of misfolded proteins.^48^, ^49^ On the other hand, resilient subjects displayed lower levels of the inflammatory marker CD68 and proteins upregulated in response to energetic or oxidative stress, including IDH1 and PINK1. These suggest healthier axons and synapses, as well as a microenvironment with less evidence of oxidative stress in the resilient individuals. PART subjects (and definite PART in particular) had similar findings to the resilient compared to ADNC subjects with dementia, including evidence of less oxidative stress (or relatively better response to oxidative stress), marked differences in proteins related to autophagy and protein degradation pathways, and evidence of better maintained synapses.

Perhaps the most interesting finding is that, at the proteomic level, the NFT‐bearing and non‐NFT‐bearing neurons in possible PART cases (which contain Aβ in the entorhinal and CA1 region) share more similarities with the NFT‐bearing and non‐NFT‐bearing neurons of ADNC than they do with definite PART (which by definition are negative for Aβ in both the neocortex and hippocampus). This demonstrates a significant role for Aβ in the development of p‐tau pathology and pathogenicity in these hippocampal subregions and suggests that the presence or absence of Aβ plays an important role in shaping the hippocampal proteome. In addition, this suggests that possible PART may fall more in the continuum of ADNC, perhaps representing a version of “early Alzheimer's disease,” while definite PART may be a completely separate entity.^38^, ^39^ We also observed that the S199 and S404 p‐tau epitopes are significantly higher in CA2 NFT‐bearing neurons in ADNC compared to definite PART, while p‐tau S396 is higher in CA2 and lower in CA1 and entorhinal cortex NFT‐bearing neurons in definite PART compared to ADNC (Figure 4 and Figure S4). This may suggest that S199 and S404 are preferentially expressed earlier in NFT development, while S396 is preferentially expressed later in NFT development.

This study is primarily limited by inherent constraints in our experimental design, the relatively small sample size and heterogeneity within diagnostic groups, and the DSP technology. Given the age range for PART and ADNC subjects and the need to study NFT‐bearing neurons, we were unable to include a separate p‐tau‐negative control cohort. While DSP provides excellent spatial resolution and quantification, the number of potential protein targets is limited by the number of antibodies used, restricting the number of possible discoveries. It is likely that there are numerous other proteomic differences between the NFT‐bearing and non‐NFT‐bearing neurons of ADNC and PART, as well as in hippocampal subregions that were unassessed using this assay. However, the coverage of general pathways (eg, oxidative stress, Aβ processing, autophagy) provides evidence for more overarching patterns of biochemical differences between these neuropathologic entities. This technology also provides future opportunities to evaluate the progression of additional Aβ‐independent tauopathies, such as chronic traumatic encephalopathy and 4R‐tauopathies (progressive supranuclear palsy and corticobasal degeneration), which also differ from ADNC in their specific patterns of hippocampal p‐tau deposition.^25^, ^63^, ^64^ In addition, future studies could include an expanded profile of proteins investigated by using mass spectrometry on microdissected hippocampal neurons.^65^, ^66^, ^67^

These data emphasize the importance of Aβ deposition in the development of hippocampal p‐tau pathology, neuronal/synaptic health, and the biologic pathways altered in and around neurons. It remains unclear why Aβ deposition appears to be necessary for p‐tau to progress from the hippocampus to the neocortex in ADNC compared to PART, but this study does strengthen the idea that Aβ and p‐tau interact in a synergistic manner, which is reflected in disease progression and ultimately cognitive symptoms. Similar to resilient individuals, definite PART subjects have altered regulation of biochemical pathways involved in oxidative stress, autophagy, misfolded protein degradation, and Aβ processing, as well as different inflammatory/microglial profile compared to ADNC. These findings advance our understanding of the mechanisms by which these disorders differ in their development and progression and provide insight for future studies aimed at understanding the pathogenesis of p‐tau‐related disorders as a whole.

## AUTHOR CONTRIBUTIONS

Conception of the work: Jamie M. Walker, Timothy E. Richardson. Design of the work: Jamie M. Walker, Miranda E. Orr, Timothy E. Richardson. Acquisition/analysis/interpretation of the data: Jamie M. Walker, Miranda E. Orr, Timothy C. Orr, Emma L. Thorn, Thomas D. Christie, Raquel T. Yokoda, Meenakshi Vij, Alexander J. Ehrenberg, Gabriel A. Marx, Andrew T. McKenzie, Justin Kauffman, Enna Selmanovic, Thomas Wisniewski, Eleanor Drummond, Timothy E. Richardson. Obtained materials and cases: Jamie M. Walker, Timothy C. Orr, Charles L. White, John F. Crary, Kurt Farrell, Tiffany F. Kautz, Elena V. Daoud, Timothy E. Richardson. Obtained funding for the work: Jamie M. Walker, Timothy E. Richardson. Drafted the work or substantially revised it: Jamie M. Walker, Tiffany F. Kautz, Elena V. Daoud, Timothy E. Richardson. All authors read and approved the final manuscript.

## CONFLICT OF INTEREST STATEMENT

Preliminary results of the data presented in this paper were published in abstract form for the 2023 American Association of Neuropathologists and 2023 Alzheimer's Association International Conference. The authors declare that they have no competing interests, conflicts of interest, or other relevant disclosures. Author disclosures are available in the supporting information.

## Supporting information



Supporting Information

Supporting Information

Supporting Information

Supporting Information

Supporting Information

Supporting Information

Supporting Information

## Data Availability

Raw data generated for this study will be made available by the corresponding authors upon request.
