# Supplementary figures and images for: Spatial proteomics of hippocampal subfield‐specific pathology in Alzheimer's disease and primary age‐related tauopathy
Source: Alzheimers Dement. 2023 Sep 30;20(2):783–97. doi: 10.1002/alz.13484 (PMC10916977; doi:10.1002/alz.13484)

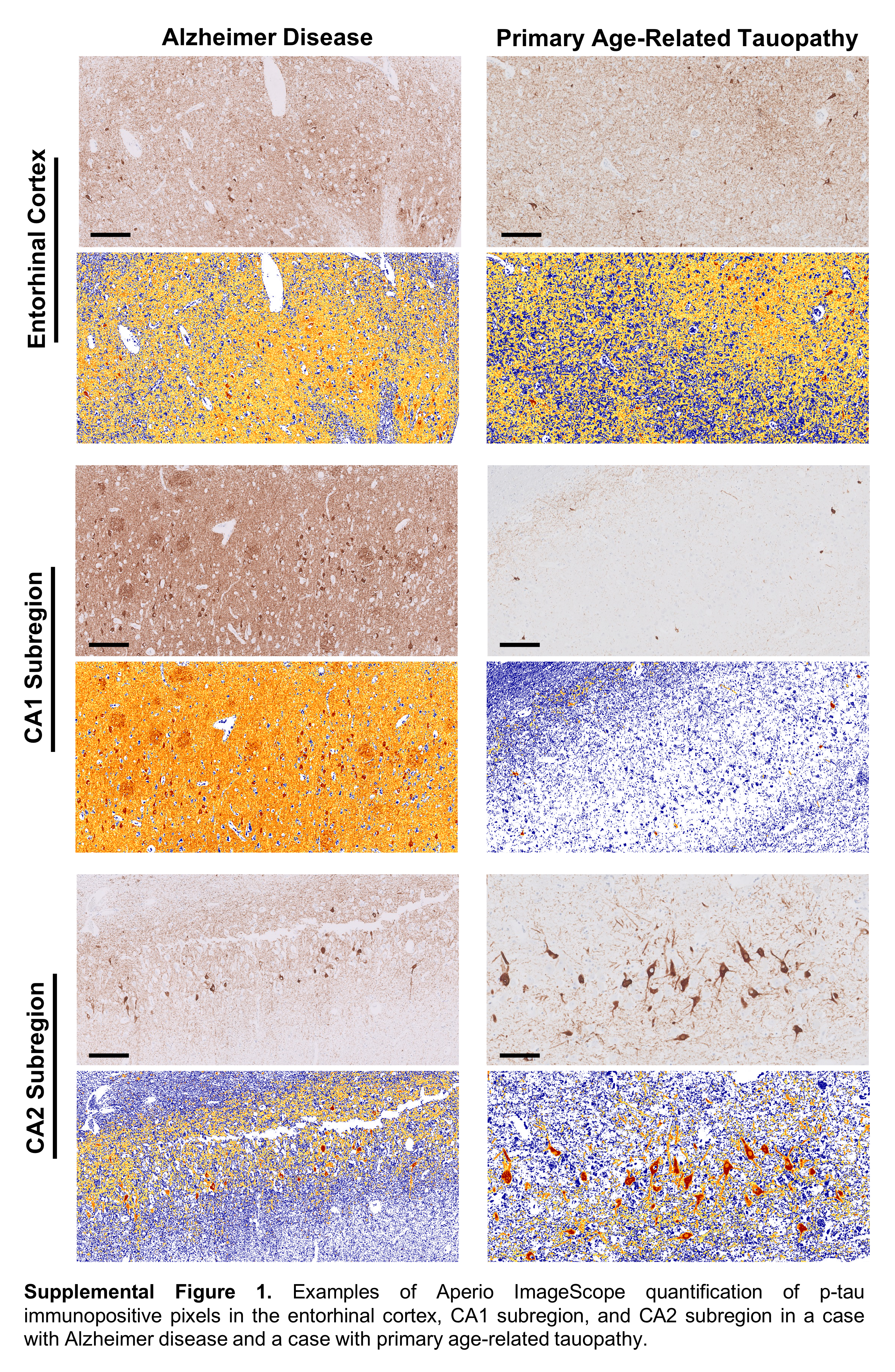

Supplement: Supplementary file 1 — Supporting Information [file ALZ-20-783-s001.tiff]

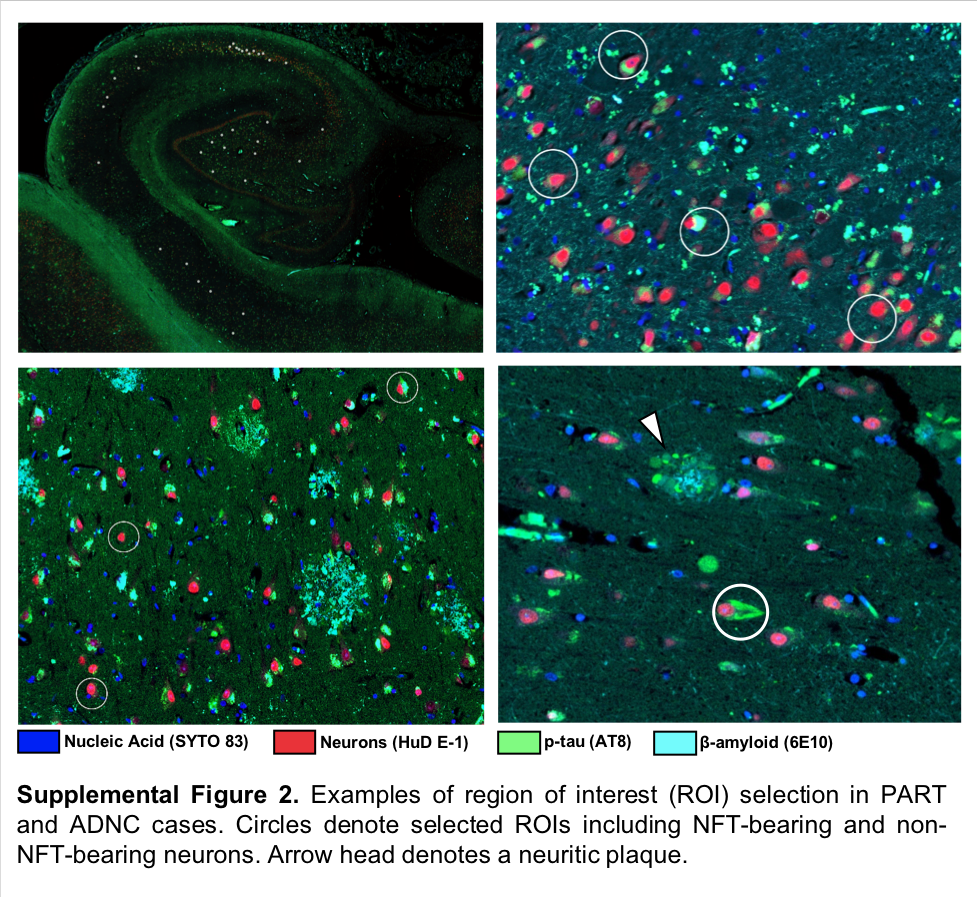

Supplement: Supplementary file 2 — Supporting Information [file ALZ-20-783-s006.tiff]

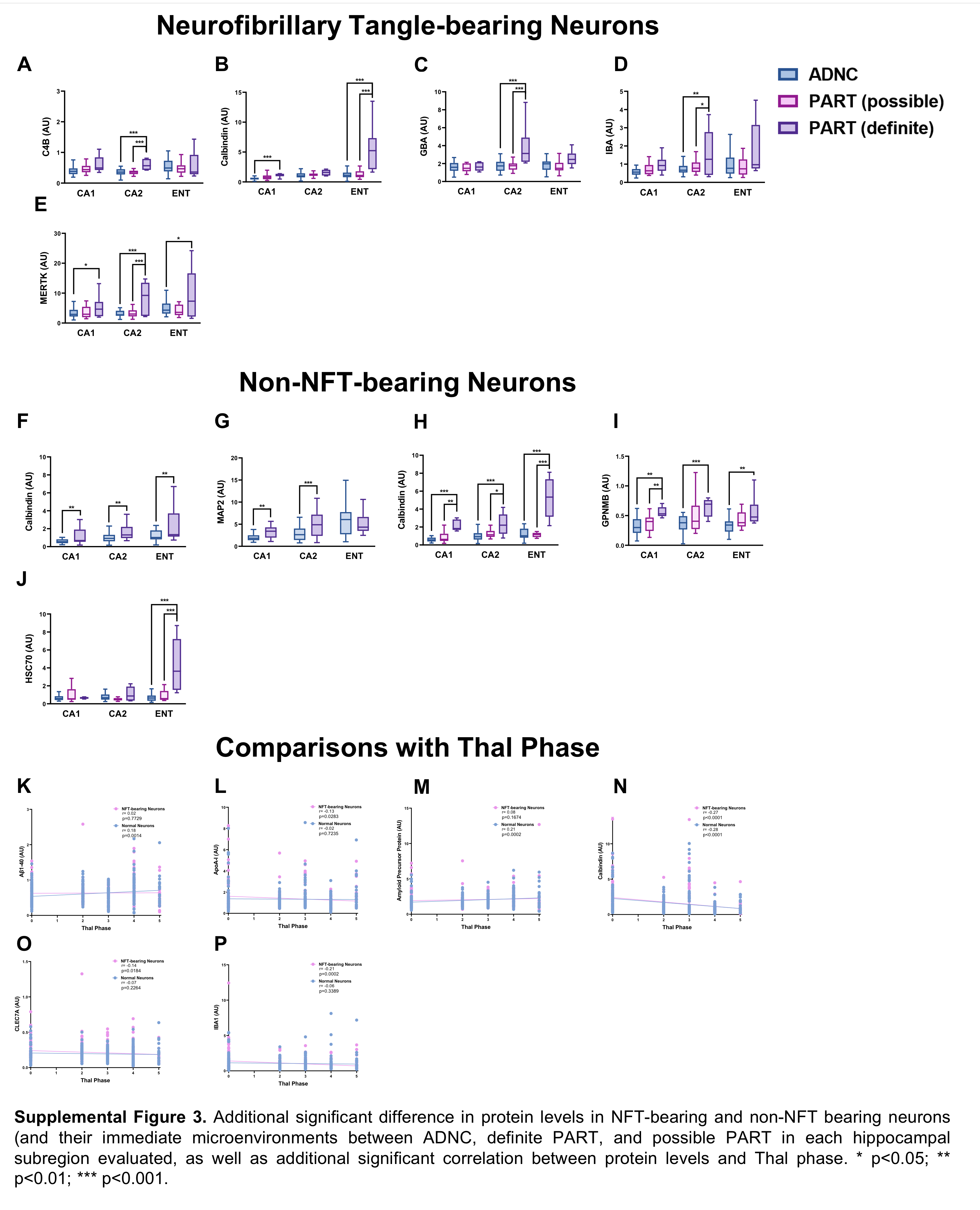

Supplement: Supplementary file 3 — Supporting Information [file ALZ-20-783-s007.tiff]

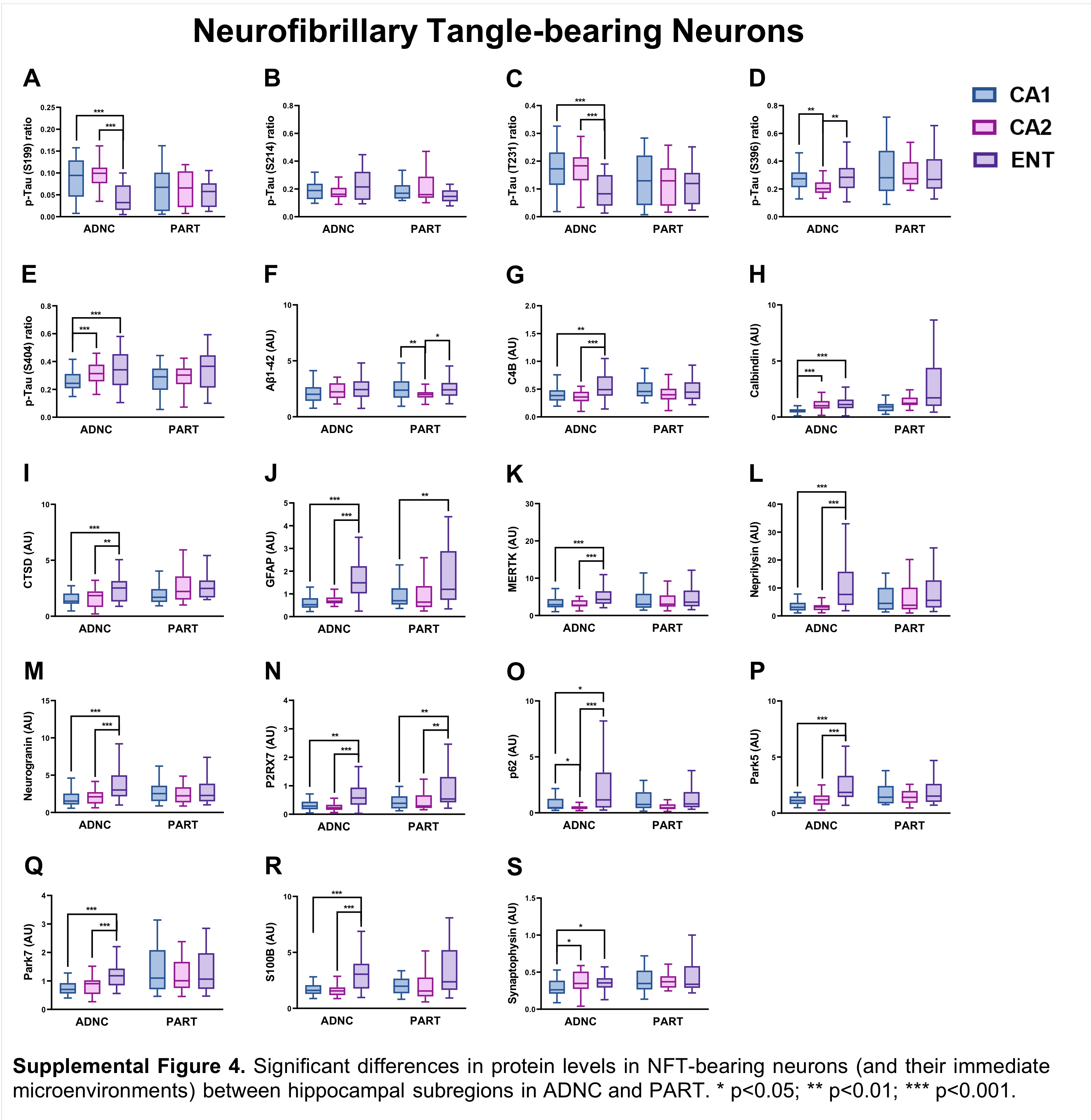

Supplement: Supplementary file 4 — Supporting Information [file ALZ-20-783-s005.tiff]

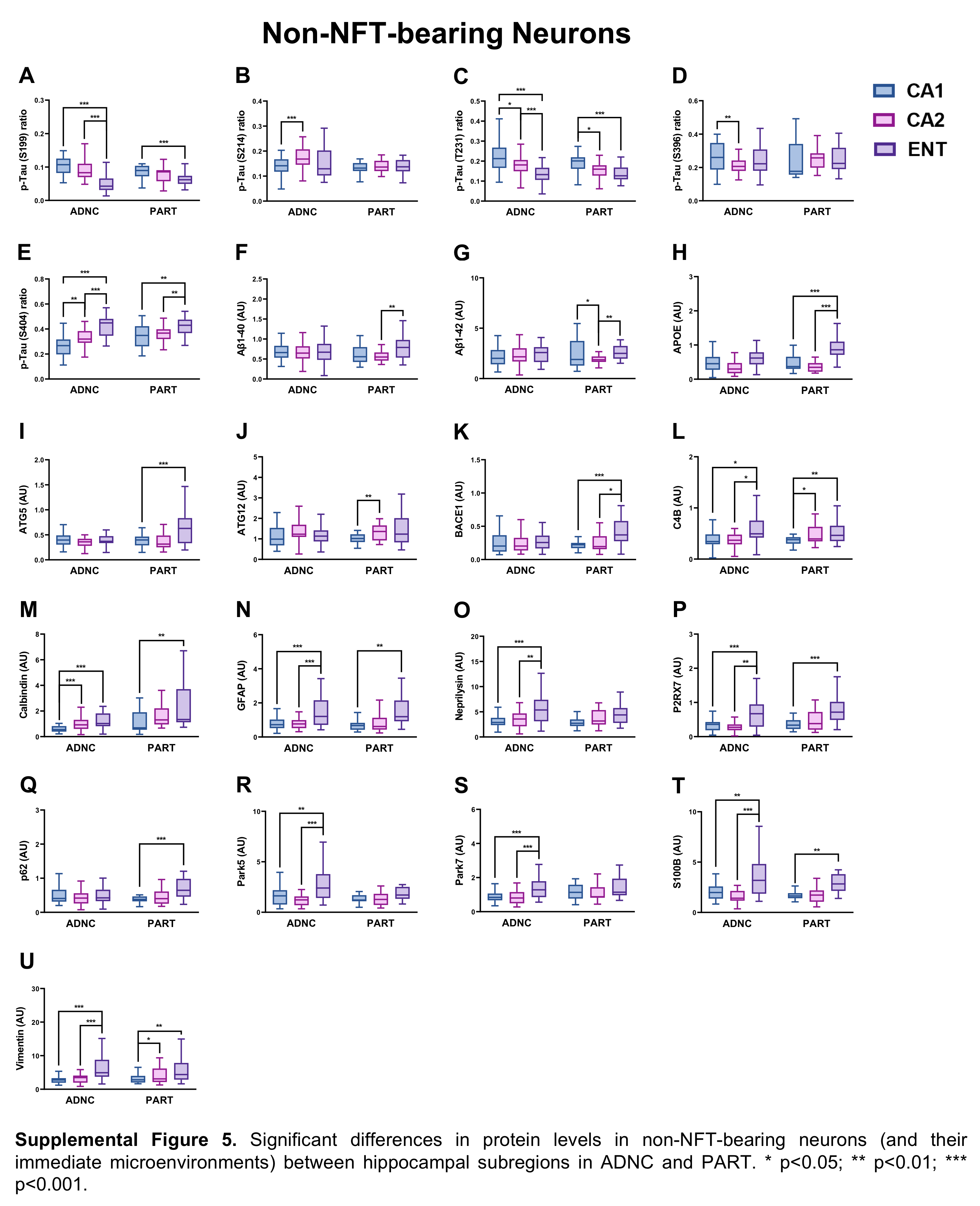

Supplement: Supplementary file 5 — Supporting Information [file ALZ-20-783-s004.tiff]
