# Supplementary material for: Spatial proteomics of hippocampal subfield‐specific pathology in Alzheimer's disease and primary age‐related tauopathy
Source: Alzheimers Dement. 2023 Sep 30;20(2):783–97. doi: 10.1002/alz.13484 (PMC10916977; doi:10.1002/alz.13484)
Supplement: Supplementary file 6 — Supporting Information [file ALZ-20-783-s003.docx]

|  |  |  |  |  |
| --- | --- | --- | --- | --- |
|  | **Supplemental Table 1.** List of 76 proteins evaluated using NanoString GeoMx DSP. | | |  |
|  | ADAM10 | FUS | p62 |  |
|  | α-synuclein | GAPDH | Park5 |  |
|  | Amyloid Precursor Protein (APP) | GBA | Park7 |  |
|  | Amyloid-β 1-40 | GFAP | Phospho-α-synuclein (S129) |  |
|  | Amyloid-β 1-42 | GPNMB | Phospho-Tau (S199) |  |
|  | ApoA-I | Histone H3 | Phospho-Tau (S214) |  |
|  | APOE | HLA-DR | Phospho-Tau (T231) |  |
|  | ATG12 | HSC70 | Phospho-Tau (S396) |  |
|  | ATG5 | IBA1 | Phospho-Tau (S404) |  |
|  | BACE1 | IDE | Phospho-TDP-43 (S409/S410) |  |
|  | BAG3 | Ki-67 | PINK1 |  |
|  | C4B | LAMP2A | PSEN1 |  |
|  | Calbindin | LC3B | Rb IgG |  |
|  | CD11b | LRRK2 | S100B |  |
|  | CD11c | MAP2 | S6 |  |
|  | CD163 | MERTK | Synaptophysin |  |
|  | CD31 | Ms IgG1 | Tau |  |
|  | CD39 | Ms IgG2a | TDP-43 |  |
|  | CD40 | Myelin basic protein (MBP) | TFEB |  |
|  | CD45 | Neprilysin | TMEM119 |  |
|  | CD68 | NeuN | Tyrosine Hydroxylase |  |
|  | CD9 | Neurofilament light (NFL) | Ubiquitin |  |
|  | CLEC7A | Neurogranin (NRGN) | Vimentin |  |
|  | CSF1R | Olig2 | VPS35 |  |
|  | CTSD | P2RX7 |  |  |
|  | EMP1 | P2ry12 |  |  |
|  |  |  |  |  |
|  |  |  |  |  |
